# Selective Recruitment of Regulatory T Cell through CCR6-CCL20 in Hepatocellular Carcinoma Fosters Tumor Progression and Predicts Poor Prognosis

**DOI:** 10.1371/journal.pone.0024671

**Published:** 2011-09-14

**Authors:** Kang-Jie Chen, Sheng-Zhang Lin, Lin Zhou, Hai-Yang Xie, Wu-Hua Zhou, Ahmed Taki-Eldin, Shu-Sen Zheng

**Affiliations:** 1 Key Laboratory of Combined Multi-organ Transplantation, Ministry of Public Health, First Affiliated Hospital, Zhejiang University School of Medicine, Hangzhou, China; 2 Department of General Surgery, Second Affiliated Hospital of Wenzhou Medical College, Wenzhou, China; New York University, United States of America

## Abstract

**Background:**

Regulatory T cells (Tregs) are highly prevalent in tumor tissue and can suppress effective anti-tumor immune responses. However, the source of the increased tumor-infiltrating Tregs and their contribution to cancer progression remain poorly understood.

**Methodology/Principal Finding:**

We here investigated the frequency, phenotype and trafficking property of Tregs and their prognostic value in patients with hepatocellular carcinoma (HCC). Our results showed that FoxP3^+^ Tregs highly aggregated and were in an activated phenotype (CD69^+^HLA-DR^high^) in the tumor site, where they can suppress the proliferation and INF-γ secretion of CD4^+^CD25^−^ T cells. These tumor-infiltrating Tregs could be selectively recruited though CCR6-CCL20 axis as illustrated by (*a*) high expression of CCR6 on circulating Tregs and their selective migration to CCR6 ligand CCL20, and (*b*) correlation of distribution and expression between tumor-infiltrating Tregs and intratumoral CCL20. In addition, we found that the number of tumor-infiltrating Tregs was associated with cirrhosis background (*P* = 0.011) and tumor differentiation (*P* = 0.003), and was an independent prognostic factor for overall survival (HR = 2.408, *P* = 0.013) and disease-free survival (HR = 2.204, *P* = 0.041). The increased tumor-infiltrating Tregs predicted poorer prognosis in HCC patients.

**Conclusions:**

The CCL20-CCR6 axis mediates the migration of circulating Tregs into tumor microenvironment, which in turn results in tumor progression and poor prognosis in HCC patients. Thus, blocking CCL20-CCR6 axis-mediated Treg migration may be a novel therapeutic target for HCC.

## Introduction

Regulatory T cells (Tregs) are a subgroup of CD4^+^ T cells characterized by expression of CD25 and a key transcription factor, known as forkhead box P3 (FoxP3) [1]. They can suppress the activation, proliferation and effector functions of various immune cells in vitro and in vivo [2]. This unique ability makes Tregs central in the prevention of autoimmune disease and maintenance of allograft tolerance. However, as a double-edged sword, Tregs can also suppress anti-cancer immune responses and favor tumor progression [3]. Thus, the relation of Tregs to carcinogenesis has become a field of intense investigation recently.

Emerging evidences demonstrate Tregs also play a central role in the immunopathogenesis of cancers [4]. First, a higher frequency of Tregs in both peripheral blood and tumors was reported in patients with a variety of cancers [4]. This list continues to grow following the current interest of studying Tregs in human tumors. Second, the number of tumor-infiltrating Tregs is negatively associated with patient prognosis [5], [6]. Third, it has been established that in murine models, selective depletion of Tregs can induce regression of established tumors [1], [7]. Overall, Treg-cell-mediated immunosuppression is one of the crucial tumor immune-evasion mechanisms and the main obstacle of successful tumor immunotherapy [1], [8], [9].

Hepatocellular carcinoma (HCC) is the fifth most common cancer worldwide [10] with a poor prognosis and limited survival in the majority of patients. Nowadays, Tregs are being extensively studied in human HCC. Increased number of Tregs has been reported in peripheral blood and, particularly, tumor tissues of patients with HCC [11], [12], [13], [14]. Furthermore, the main mechanisms by which Tregs facilitate liver carcinogenesis are to prevent CD8^+^ T cells from proliferating in response to tumor-associated antigens and from becoming cytotoxic effector cells [12], [13], [14]. However, little is known about the mechanisms leading to the increased Tregs in tumor tissue. It is summarized that there are four mechanisms responsible for this, and the recruitment of Tregs is the most important approach [1].

The migration of lymphocytes to the target site was a multi-step procedure, in which signals from chemokines/chemokine receptors play a critical role [15]. The migratory capacity of Tregs is controlled by distinct signals from chemokines/chemokine receptors [4]. The following chemokine receptors have been reported on Tregs: CCR2, CCR4, CCR5, CCR6, CCR7, CCR8, CXCR3, and CXCR4 [4], [16], [17], [18]. In human ovarian and breast cancers, the CCL22/CCR4 signal has been demonstrated to mediate this procedure [5], [19]. However, the migratory determinants for Treg-cell migration into tumor tissues of HCC patients remain unknown. In this study, we found that the CCR6-CCL20 axis determines the migration of circulating Tregs into tumor tissues in HCC patients.

## Results

### CD4^+^CD25^+^FoxP3^+^ Tregs are highly enriched in tumors of HCC patients

**Figure 1:**
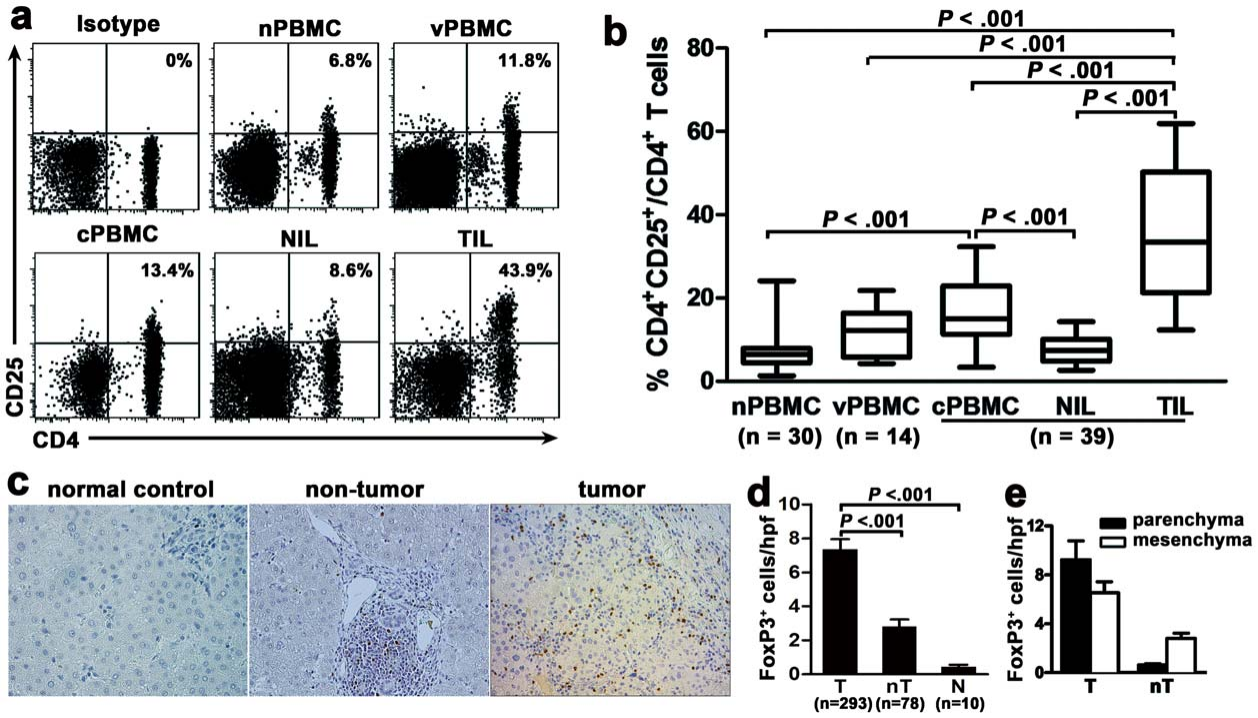
CD4^+^CD25^+^FoxP3^+^ Tregs are highly enriched in tumors of HCC patients. Tregs (CD4^+^CD25^+^ T cells) were gated from CD3^+^ T cells by flow cytometry. (a, b) Representative plots (a) and statistical analysis (b) show that the frequency of CD4^+^CD25^+^ T cells was higher in HCC patients, especially among TIL. The percentages in (a) represent the frequency of CD4^+^CD25^+^ T cells among CD4^+^ T cells. The data in (b) are expressed in box plots, in which the horizontal lines illustrate the 25th, 50th and 75th percentiles. (c, d) Representative images (c) and statistical analysis (d) of immunohistochemical staining of FoxP3^+^ lymphocytes in the tumor and non-tumor tissue from HCC patient and normal control liver. Magnification, ×200. The data in (d) show that the number of FoxP3^+^ cells is significantly higher in tumor than in non-tumor or normal control. hpf, high-powered field. (e) Statistical analysis shows FoxP3^+^ cells preferentially aggregate in the parenchyma of tumors.

We initially investigated whether Tregs accumulate in tumors of HCC patients. The prevalence of Tregs was identified by flow cytometry after cell surface labeling of CD4 and CD25 molecules. As expected, the frequency of CD4^+^CD25^+^ T cells in tumor-infiltrating lymphocyte (TIL), representing 34.9±2.7% of CD4^+^ T cells, was significantly higher than those in non-tumor-infiltrating lymphocyte (NIL; 7.8±0.6%), and peripheral blood mononuclear cells from HCC patients (cPBMC; 16.7±1.2%), HBV patients (vPBMC; 11.7±1.3%) and healthy donors (nPBMC; 6.7±0.7%) (*P*<0.001, Figure 1a, b). Additionally, the frequency of circulating CD4^+^CD25^+^ T cells was also higher in HCC patients compared with healthy donors (*P*<0.001).

**Figure 2:**
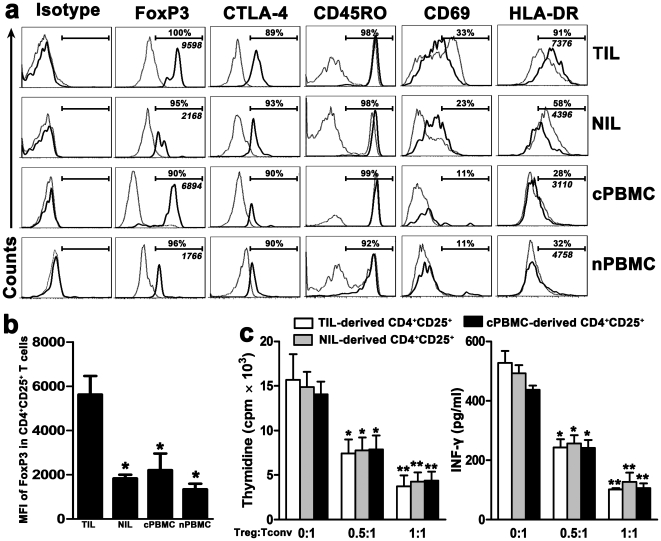
Phenotypic and functional analysis of CD4^+^CD25^+^ Tregs in HCC patients. (**a**) Representative FoxP3, CTLA-4, CD45RO, CD69 and HLA-DR expression profiles in CD4^+^CD25^+^ (thick line) and CD4^+^CD25^−^ (dotted line) T cells from the four studied groups. Specific isotypes were used as negative control. The percentages represent the frequencies of various markers on CD4^+^CD25^+^ T cells. The italic numerical values represent MFI of FoxP3 in CD4^+^CD25^+^ T cells. (b) Statistical analysis shows that the MFI of FoxP3 in CD4^+^CD25^+^ T cells from TIL was significantly higher than those from NIL, cPBMC, and nPBMC. *P<0.05, compared with TIL. (c) Purified CD4^+^CD25^+^ T cells (Tregs) from TIL, NIL, and cPBMC could similarly inhibit the proliferation and INF-γ production of autologous CD4^+^CD25^−^ T cells (Tconv) in a dose-dependent manner (n = 3 for each group). *P<0.05; **P<0.01, compared with autologous controls (Treg∶Tconv = 0∶1). Data in (b) and (c) represent the mean±S.E.M.

To determine quantity and distribution of Tregs in tumor tissue, we performed immunohistochemical staining of FoxP3 in paraffin-embedded tissue from HCC patients (Group 1, n = 293). Consistent with the flow cytometric analysis, we observed that FoxP3^+^ cells preferentially accumulated in tumor tissue (7.3±0.6/hpf) rather than in non-tumor tissue (2.8±0.4/hpf) or normal control livers (0.4±0.1/hpf) (both *P*<0.001 for T vs. nT and T vs. NC; Figure 2c, d). Importantly, the distribution of FoxP3^+^ cells in tumor region differed from that in non-tumor region. In tumor tissue, most of FoxP3^+^ cells locate in the parenchymal region, where the FoxP3^+^ cells are close to liver tumor cells, while in non-tumor tissue, the majority of FoxP3^+^ cells aggregate in the mesenchymal region (parenchyma: mesenchyma, 7∶5 in tumor vs. 5∶22 in non-tumor, *P*<0.001; Figure 1e). These results suggest that Tregs are highly enriched in tumor tissue, and that physical contact between Tregs and tumor cells may be necessary to mediate regulatory functions [11].

### Phenotypic and functional analysis of CD4^+^CD25^+^ Tregs in HCC patients

CD4^+^CD25^+^ T cells, whatever their origin, highly expressed FoxP3, cytolytic T lymphocyte-associated antigen 4 protein (CTLA-4) and CD45RO, three specific markers constitutively expressed by classic Tregs (Figure 2a) [20]. However, the mean fluorescence intensity (MFI) of FoxP3 in CD4^+^CD25^+^ T cells from TIL was higher than those from NIL, cPBMC, and nPBMC (*P*<0.05, Figure 2b). Importantly, most CD4^+^CD25^+^ Tregs from TIL and NIL exhibited an activated memory phenotype (CD69^+^HLA-DR^+^), with the highest CD69 and HLA-DR expression on TIL-derived CD4^+^CD25^+^ Tregs (Figure 2a). We also found that CD4^+^CD25^+^ T cells from TIL, NIL, or cPBMC similarly inhibited the proliferation and INF-γ production of autologous CD4^+^CD25^−^ T cells (Tconv) in a dose-independent manner (n = 3, *P*<0.05; Figure 2c). These data indicated that CD4^+^CD25^+^ T cells from tumor tissue of HCC have an activated phenotype and immunosuppressive property.

### Increased CCL20, secreted by liver tumor cells, is associated with the number of tumor-infiltrating Tregs

**Figure 3:**
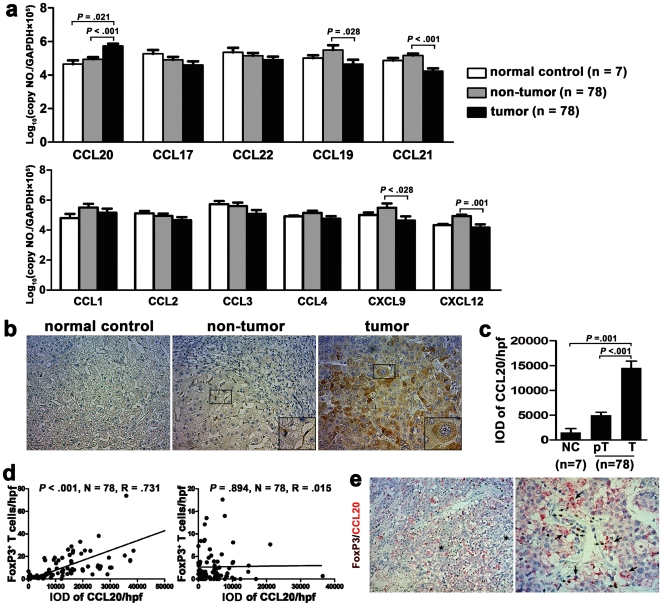
Increased CCL20, secreted by liver tumor cells, is associated with the number of tumor-infiltrating Tregs. (a) Real-time PCR of selective chemokines in tumor and non-tumor regions of HCC patients and in normal control liver. The value was normalized to GAPDH, multiplied by 10^5^, and log transformed. (b) immunohistochemical staining of chemokine CCL20 (brown, cytoplasm). Numerous CCL20-secreting tumor cells are seen in tumor region, and a few CCL20-secreting Kuffer cells in non-tumor region. Magnification, ×200; insert boxes, ×400. (c) Statistical analysis shows the CCL20 level in tumor tissue is significantly stronger than that in non-tumor and normal liver tissues. IOD, integrated optical density. (d) The correlations between CCL20 level and the number of FoxP3^+^ cells in tumor (left) and non-tumor (right) regions. The expression of CCL20 and FoxP3 were detected by immunohistochemical staining, and quantified as described in “Materials and Methods”. (e) Double staining of CCL20 (red, cytoplasm) and FoxP3 (brown, nucleus). The regions expressing high and low levels of CCL20 are marked by “★” and“*”, respectively. Big arrows indicate CCL20-secreting tumor cells, and small arrows FoxP3^+^ cells. Magnification, ×200, left; ×400, right.

To identify what chemokine mediates the recruitment of tumor-infiltrating Tregs, we investigated the selective chemokines in tumor and non-tumor regions by real-time PCR. These chemokines are ligands of CCR2, CCR4, CCR5, CCR6, CCR7, CCR8, CXCR3, and CXCR4, all of which have been reported to be expressed on Tregs [21]. We found that only the mRNA expression of CCL20 was significantly increased in tumor than in non-tumor and normal control tissues (T vs. nT, *P*<0.001; T vs. NC, *P* = 0.021; Figure 3a). For the other chemokines, their mRNA expression was lower in tumor than in non-tumor region (Figure 3a). Notably, the expression of CCL19, CCL21, CXCL9, and CXCL12 was significantly decreased (*P*<0.05).

Next, we detected the CCL20 expression at the protein level by immunohistochemical to verify the results of PCR, and to clarify the source of CCL20. Consistent with real-time PCR, the CCL20 expression in tumor tissue was significantly stronger than that in non-tumor (integrated optical density/hpf, 14529 vs. 4969, *P*<0.001) and normal liver tissue (vs. 1534, *P* = 0.001) (Figure 3b, c). We observed large amounts of CCL20-secreting cancer cells and, occasionally, scattered CCL20-secreting Kuffer cells in tumor region (Figure 3b, right). However, in non-tumor region, CCL20 is secreted mainly by Kuffer cells (Figure 3b, middle) [22]. These results collectively indicate that CCL20, rather than other chemokines, mediates the trafficking of Tregs into tumor tissue; and that liver tumor cells are major sources of CCL20 in tumor microenvironment.

Furthermore, we evaluated the relationship of Tregs to CCL20 in the same tumor environment by immunohistochemistry. First, a strongly positive correlation was observed between the number of FoxP3^+^ Tregs and CCL20 expression in tumor tissue (*P*<0.001; Figure 3d, left), but not in non-tumor tissue (*P* = 0.894; Figure 3d, right). Next, we performed immunohistochemical double-staining of FoxP3 and CCL20 to visualize the mutual distributions of Tregs and CCL20. As shown, FoxP3^+^ cells mainly assemble in the sites where CCL20 is highly expressed (Figure 3e, left). Meanwhile, we observed the attraction of tumor cells to FoxP3^+^ cells by secreting massive CCL20 (Figure 3e, right). These results indicate that, to some extent, the amount of tumor-secreted CCL20 determines the prevalence of Tregs in the same site.

### Circulating CD4^+^CD25^+^ Tregs in HCC patients highly express CCR6, and selectively migrate to tumors under recruitment of CCL20

**Figure 4:**
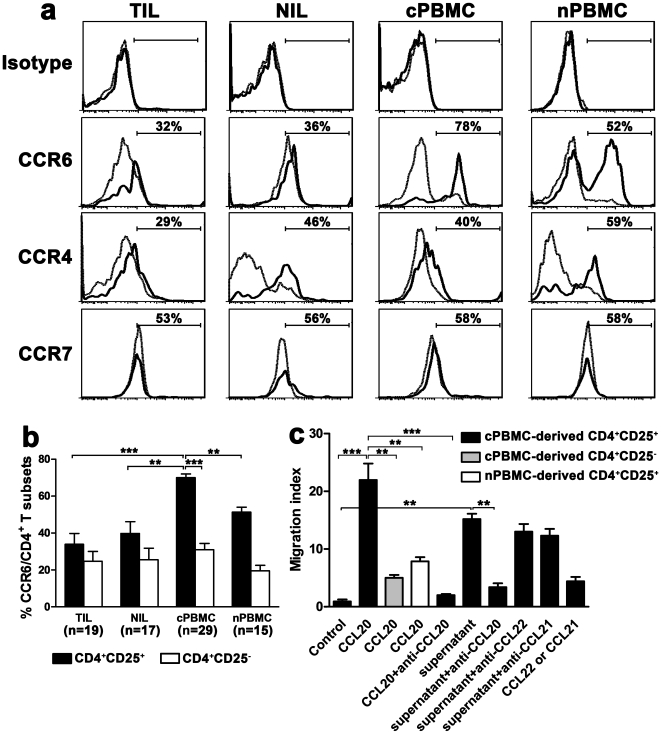
Circulating CD4^+^CD25^+^ Tregs in HCC patients highly express CCR6, and selectively migrate to tumors under recruitment of CCL20. (a) Representative plots of CCR6, CCR4 and CCR7 on CD4^+^CD25^+^ (solid line) and CD4^+^CD25^−^ (dotted line) T cells from different compartments. Specific isotypes were used as negative control. The percentages represent frequencies of CCR6, CCR4 and CCR7 on CD4^+^CD25^+^ T cells. (b) Statistical analysis shows that cPBMC-derived CD4^+^CD25^+^ Tregs expressed significantly higher CCR6 than autologous CD4^+^CD25^−^ T cells or Tregs from the other groups. **P<0.01, ***P<0.001. (c) CD4^+^CD25^+^ T cells migrate in response to recombinant human CCL20 or supernatant of SMMC-7721 cells (n = 3). A specific antibody to CCL20 significantly inhibits CD4^+^CD25^+^ T cell migration. Data represent the mean±S.E.M. **P<0.01, ***P<0.001.

To reveal whether the expression of CCR6, the exclusive receptor of CCL20, on circulating Tregs was increased correspondingly, we analyzed the expression of CCR6 on CD4^+^ T-cell subsets by flow cytometry. We found cPBMC-derived CD4^+^CD25^+^ Tregs expressed significantly higher CCR6 (70±2%, n = 29) than autologous CD4^+^CD25^−^ T cells or Tregs from the other groups (*P*<0.01 or 0.001, Figure 4a, b). Different from CCR6 expression, the frequency of CCR4 on cPBMC-derived Tregs (40±3%, n = 20) was lower than that on nPBMC-derived counterparts (55±3%, n = 12; *P*<0.001), though higher than that on autologous CD4^+^CD25^−^ T cells (15±2%, *P*<0.001) (Figure 4a). CCR7 was similarly expressed on both CD4^+^CD25^+^ and CD4^+^CD25^−^ T cells from blood or tissue (Figure 4a). Altogether, these observations suggest that Tregs in peripheral blood of HCC patients preferentially express high frequency of CCR6.

Next, we detected the chemotaxis of cPBMC-derived CD4^+^CD25^+^ T cells to CCL20 in vitro. Significant chemotacitc responses to recombinant human CCL20 were observed in cPBMC-derived CD4^+^CD25^+^ T cells than in autologous CD4^+^CD25^−^ T cells and cPBMC-derived counterparts (n = 3, *P*<0.01, Figure 4c). Further, a neutralizing monoclonal antibody to CCL20 did markedly blocked CCL20-induced migration of cPBMC-derived CD4^+^CD25^+^ T cells (*P*<0.001, Figure 4c). In contrast to CCL20, both CCL22 and CCL21 (specific ligands for CCR4 and CCR7, respectively) only induced weak migration of cPBMC-derived CD4^+^CD25^+^ T cells. SMMC-7721, a human HCC cell line, was detected to highly secrete CCL20 by real-time PCR and enzyme-linked immunosorbent assay (data not shown). The chemotactic effect of SMMC-7721 supernatant was also tested on cPBMC-derived CD4^+^CD25^+^ T cells. Like CCL20, SMMC-7721 supernatant induced significant migration of cPBMC-derived CD4^+^CD25^+^ T cells (*P*<0.01 versus control), which can be efficiently blocked by antibody to CCL20, but not by antibody to CCL22 or CCL21 (Figure 4c). Thus, CCL20 mediates Tregs trafficking in vitro and may recruit Tregs into the tumor.

**Figure 5:**
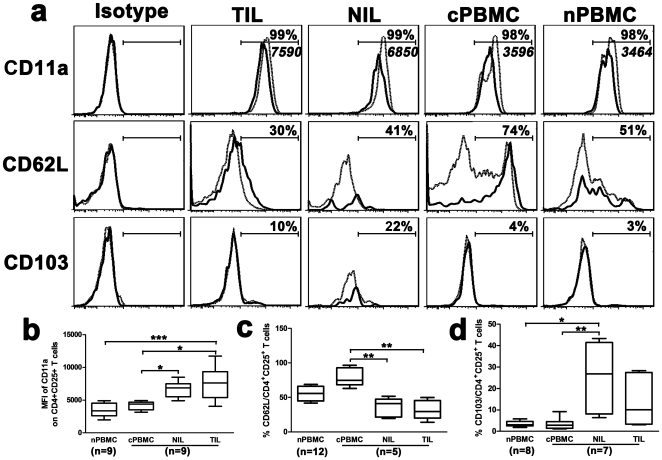
Differential expression of CD11a, CD62L and CD103 on CD4^+^CD25^+^ Tregs. (a) Representative CD11a, CD62L, and CD103 expression profiles on CD4^+^CD25^+^ (solid line) and CD4^+^CD25^−^ (dotted line) T cells from the four studied groups. Specific isotypes were used as negative control. The percentages represent the frequencies of various markers on CD4^+^CD25^+^ T cells. The italic numerical values represent MFI of CD11a on CD4^+^CD25^+^ T cells. (b–d) Statistical analyses of CD11a intensity (b), as well as CD62L (c) and CD103 (d) frequencies on CD4^+^CD25^+^ Tregs. The data are expressed in box plots, in which the horizontal lines illustrate the 25th, 50th and 75th percentiles. *P<0.05, **P<0.01, ***P<0.001.

Furthermore, we investigated the expression of integrins LFA-1 (CD11a) and αEβ7 (CD103), and L-selectin (CD62L) on Tregs, since the combination of selecins, integrins and chemokine receptors is responsible for the selective migration of Tregs [23], [24]. We found that nearly all CD4^+^CD25^+^ T cells expressed CD11a (>98%, Figure 5a), a dominant integrin involved in lymphocyte arrest [25]. Importantly, the intensity of CD11a on TIL-derived CD4^+^CD25^+^ T cells (MFI, 7455±805) was significantly stronger than those on cPBMC- (4144±229) or nPBMC-derived counterparts (3480±364) (*P*<0.05, Figure 5b), suggesting that CD11a might be helpful to Treg migration. The expression of CD62L, essential molecular for homing to lymph node [4], was strongly reduced in TIL-derived Tregs compared with patient blood Tregs (*P*<0.01, Figure 5a, c), while that of CD103, critical to skin-specific homing [21], was extremely low in all studied groups (<25%; Figure 5a, d). Thus, we consider that neither CD103 nor CD62L might be related to liver-specific migration of Tregs.

### Treg prevalence is associated with tumor progression and predicts poor survival of HCC patients

**Figure 6:**
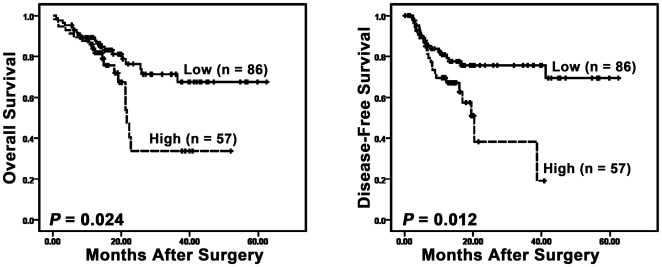
Accumulation of tumor Treg cells predicts poor survival in patients with hepatocellular carcinoma. The cumulative overall survival and disease-free survival of HCC patients were estimated using the Kaplan-Meier method and compared by the log-rank test. Patients with high FoxP3^+^ Tregs (dashed lines) had significantly poorer survival compared with individuals with low FoxP3^+^ Treg density (solid lines).

Finally, we assessed the association of tumoral FoxP3^+^ Tregs with clinical characteristics. Using the median number of FoxP3^+^ Tregs determined by immunohistochemical staining (6.6 cells/hpf) as the cutoff, patients were divided into two groups: High (n = 148) and Low Treg groups (n = 145). We found that increased tumor FoxP3^+^ cells were correlated with the presence of cirrhosis (*P* = 0.011) and poorer tumor differentiation (*P* = 0.003) (Table 1), indicating that the accumulation of these cells is associated with disease progression. In that case, we further predicted whether tumor Tregs would adversely affect survival. To test this prediction, we analyzed all relevant clinical and pathological information, including the number of tumor Tregs, with tumor-associated survival on 143 patients with follow-up evaluation. There was a significant inverse correlation of tumor Treg density with both overall survival (median survival time, 28 months in High vs. 48 months in Low, *P* = 0.024) and disease-free survival (median survival time, 22 months in High vs. 47 months in Low, *P* = 0.012) (Figure 6). Using multivariable analysis, tumor FoxP3^+^ Treg density was found to be an independent prognostic factor of overall survival (Hazard Ratio, 2.4; *P* = 0.013) and disease-free survival (Hazard Ratio, 2.0; *P* = 0.041) (Table 2). These results show that the prevalence of tumor FoxP3^+^ Tregs can serve as an independent predictor of poor prognosis for HCC patients.

**Table 1:** Correlation between tumor FoxP3^+^ cell and clinicopathologic characteristics.

	FoxP3^+^ Cells		
Characteristics	Low	High	*P*
No. of Patients	148	145	
Age, ≤50/>50 years	63/85	54/91	0.325
Sex, Male/Femal	128/20	118/27	0.234
HBsAg, Negative/Positive	27/121	31/114	0.501
AFP level, ≤400/>400 ng/mL	97/51	81/64	0.090
ALT level, ≤40/>40 U/L	75/73	87/58	0.108
Child-Pugh score, A/B+C	136/12	131/14	0.520
Liver cirrhosis, Yes/No	100/48	77/68	0.011
Tumor size, ≤5/>5 cm	80/68	75/70	0.690
Tumor encapsulation, None/Presence	117/31	123/22	0.199
Tumor number, Single/Multiple	103/45	102/43	0.889
Vascular invasion, Yes/No	39/109	41/104	0.721
TNM stage, I+II/III+IV	104/44	98/47	0.620
Tumor differentiation, I−II/III	89/59	62/83	0.003

HBsAg, Hepatitis B surface antigen; AFP, α-fetoprotein; ALT, alanine aminotransferase.

**Table 2:** Univariate and Multivariate analysis of factors associated with survival and recurrence.

	Overall survival				Disease-free survival			
	Univariate		Multivariate*		Univariate		Multivariate*	
Variables	HR	*P*	HR	*P*	HR	*P*	HR	*P*
Age, ≤50/>50 years	1.287	0.448			0.445	0.012	0.448	0.035
Sex, Male/Femal	2.262	0.129			0.995	0.991		
HBsAg, Negative/Positive	0.644	0.269			0.801	0.594		
AFP, ≤400/>400 ng/mL	2.472	0.007	NA	0.061	1.816	0.067	NA	0.650
ALT, ≤40/>40 U/L	1.501	0.226			1.383	0.317		
Child-Pugh score, A/B+C	1.022	0.957			0.682	0.390		
Liver cirrhosis, Yes/No	0.712	0.366			0.936	0.858		
Tumor size, ≤5/>5 cm	2.032	0.032	2.246	0.020	1.778	0.067	NA	0.642
Tumor encapsulation, None/Presence	0.036	0.064	NA	0.097	0.096	0.120		
Tumor number, Single/Multiple	0.627	0.152			0.462	0.014	NA	0.116
Vascular invasion, Yes/No	2.247	0.013	NA	0.252	2.139	0.018	NA	0.985
TNM stage, I+II/III+IV	1.883	0.052	NA	0.363	4.169	0.000	3.723	0.000
Tumor differentiation, I−II/III	1.760	0.095	NA	0.669	1.983	0.037	NA	0.230
FoxP3, Low/High	2.084	0.027	2.408	0.013	2.204	0.14	2.204	0.041

Univariate and multivariate analysis: Cox proportional hazards regression model.

HR, Hazard Ratio; NA, not adopted.

*Multivariate variables were adopted by univariate analysis (*P*<0.10).

## Discussion

In this study, we show that the presence of tumor-infiltrating CD4^+^CD25^+^ T cells are typical Tregs based on their CD4^+^CD25^+^FoxP3^high^CD45RO^+^CTLA-4^+^ phenotype and suppressive functions. We also found that FoxP3^+^ Tregs were concentrated within HCC tumors, and that the intratumoral prevalence of FoxP3^+^ Tregs was associated with disease progression and poor prognosis. More importantly, we revealed that the CCL20-CCR6 axis controls the migration of circulating Tregs into tumor, resulting in the increased frequency and quantity of Tregs in tumor tissue.

It is reported that CD4^+^CD25^+^ T cells (Tregs) constitute 5–10% of peripheral CD4^+^ T cells in healthy people [1], [26]. Consistent with these reports, the CD4^+^CD25^+^ T cells counted for 6.7±0.7% of CD4^+^ T cells in healthy donor based on our gating strategy. Importantly, we observed a substantial increase of tumor-infiltrating CD4^+^CD25^+^ Tregs in both frequency and quantity, in agreement with previous findings [11], [13], [27]. FoxP3 is a master regulator of Tregs for their development, maintenance and function [28]. Here, we found that the level of FoxP3 was higher in tumor-derived Tregs. However, Tregs from HCC patients showed similar immunosuppressive effects, as CD4^+^CD25^+^ T cells from either TIL, NIL or cPBMC similarly inhibited the proliferation and INF-γ production of autologous CD4^+^CD25^−^ T cells. These data suggested that the prevalence, rather than the superior suppressive activity, of Tregs in the tumor microenviroment results in reduced tumor-specific immunity [1]. In addition, we showed that, in contrast to paired blood Tregs, tumor-infiltrating Tregs display an activated phenotype, as they expressed elevated levels of CD69 and HLA-DR. Only ∼30% of Tregs expressed low level of HLA-DR in patient peripheral blood, while the majority of tumor-derived Tregs highly expressed HLA-DR, which identifies a terminally differentiated subpopulation of effector Tregs [3]. Therefore, this means that most of Tregs are terminally differentiated ones in tumor microenvironment.

The high number of tumor-infiltrating in the liver tumor environment raises the question of their recruitment. Over the past decade, CCR4 and its ligands have been demonstrated to play critical role in recruiting circulating Tregs into tumor tissue. Circulating Tregs have been revealed to express high CCR4 levels and to selectively migrate in response to CCR4 ligands produced in the tumor microenvironment [5], [19], [29], [30]. However, we observed that, in HCC patients, circulating Tregs highly express CCR6 and migrate to CCL20 present in the tumor microenvironment. This conclusion is based on two findings. First, only the CCR6 ligand CCL20 had elevated expression at both mRNA and protein levels in tumor tissues. Moreover, the CCL20 expression was strong correlated with the number of FoxP3^+^ Tregs in tumor environment. Second, the circulating Tregs from HCC patients highly expressed CCR6, and selectively migrate in response to CCL20 in vitro. It is worth noting that the Tregs in tumor environment expressed low to even undetectable CCR6. We infer this may result from the strong expression of CCL20 in tumor environment, which inversely internalizes the CCR6 expression like CCR4 [19]. In the past, CCR6 was mainly implicated to be responsible for the inflammatory recruitment of Tregs. Fox example, CCR6 is essential for the optimal recruitment of Tregs to sites of Th17-mediated inflammation in experimental autoimmune encephalomyelitis (EAE) [31]. However, recently, accumulating finding show that the CCR6 expression on Tregs also plays a critical role in tumor development [32], [33]. It is well accepted that not only the suppressor potential but also appropriate localization determines the in vivo suppressive capacity of Tregs [34]. All these results, together with our data, show an important role of CCR6 in Treg-mediated immunosuppression.

Although they are critical factors to mediate Treg migration into tumors or lymph nodes, CCR4 and CCR7 are at least not essential for migration of circulating Tregs from HCC patients in this study. First, none of the ligands for CCR4 and CCR7 had enhanced expression in tumor environment. Second, although they had much higher expression of CCR4 than CD4^+^CD25^−^ T cells, the circulating Tregs appeared to have significantly lower frequency of CCR4 than their counterparts in normal controls. Likewise, the expression of CCR7 between CD4^+^ T subsets was similar and did not fluctuate substantially among groups. Third, chemotaxis assays failed to show selective migration of circulating Tregs from HCC patients to CCL22 and CCL21.

Several studies assessed the association of increased tumor-infiltrating Tregs with clinical characteristics and revealed different results. Tang et al. found that high tumor Treg density was associated with both absence of tumor encapsulation and presence of tumor vascular invasion [6]. Another studies revealed that the prevalence of Tregs was correlated with the presence of cirrhosis and later TNM stages [35]. We found that increased tumor FoxP3^+^ Tregs was also correlated with cirrhosis background, but more importantly with poorer tumor differentiation. Though somewhat different, all these results mean Tregs contribute to tumor progression in HCC patients. Meanwhile, elevated tumor-infiltrating Treg number (>6.6/hpf) also predicted a poorer prognosis with shorter disease-free and overall survival, in line with previous reports in liver tumors [6], [35].

In conclusion, we report here the frequency, phenotype, and trafficking property of Tregs and their correlation with clinicapathologic factors in HCC patients. Especially, we found that CCR6 is a liver-specific determinant for the trafficking of circulating Tregs into tumor. These results extend our understanding of the mechanism of liver carcinogenesis. Thus, apart from depleting Tregs, blocking Treg-cell trafficking into tumor represents a potential strategy for treating human HCC.

## Methods

### Ethics Statement

The study protocol was approved by the Institutional Review Board of Key Lab of Combined Multi-organ Transplantation, Ministry of Public Health. Informed written consent was obtained from patients according to the Declaration of Helsinki.

### Subjects

A total of 293 patients with HCC were enrolled in this study. None of the HCC patients had received immunosuppressive drugs or chemotherapy. Paraffin-embedded, formalin-fixed liver sections were obtained from 293 patients (Group 1) who underwent surgical resection between 2005 and 2010. Of these patients, 143 patients with follow-up evaluation (Group 2) were further enrolled in survival analysis. Overall survival was defined as the interval between the dates of surgery and death, and Disease-free survival the interval between the dates of surgery and recurrence or the last follow-up. Matched fresh blood, tumor and non-tumor (at least 3 cm distant from the tumor tissue) samples were also obtained from 39 patients (Group 3) with HCC. The patient characteristics of three groups are described in Table 3. Liver tissues from 10 donors for living donor liver transplantation, and fresh blood samples from 14 patients with HBV infection but without HCC and 30 healthy volunteers were used as control.

**Table 3:** Characteristic of Patients with HCC.

	Group		
Variables	1	2	3
No. of patients	293	143	39
Mean age ± SD, yr	54.3±11.2	52.0±10.1	53.1±9.8
Gender, male/female	246/47	125/18	33/5
HBsAg, positive/negative	235/58	120/23	37/2
Background, CH/LC	102/139	30/105	13/26
AFP level (ng/ml), >400/≤400	115/178	58/85	11/28
Child-Pugh score, A/B/C	267/24/2	116/25/2	39/0/0
ALT level, >40/≤40 U/L	131/162	70/73	17/22
Tumor size* (cm), >5/≤5	138/155	65/78	18/21
Number of tumors, single/multiple	205/88	88/55	7/32
Tumor stage, I/II/III/IV	148/55/83/7	63/32/41/7	20/11/8/0
Tumor differentiation, I/II/III	21/130/142	6/72/65	2/21/16

HBV, hepatitis B virus; HCV, hepatitis C virus; CH, chronic hepatitis; LC, liver cirrhosis; AFP, alpha-fetoprotein.

* Tumor size is expressed by the maximum diameter of tumors.

### Isolation of peripheral blood mononuclear cells (PBMC), TIL, and NIL

PBMC were isolated by Ficoll-Hypaque (Sigma-Aldrich, St. Louis, MO) density gradient separation. TIL and NIL were isolated as described [27]. Briefly, the tissue was cut into small pieces and incubated in an enzyme mixture containing 0.05% collagenase IV (Invitrogen, Carlsbad, CA) and 0.001% DNase I (Sigma-Aldrich) for 1 h. Dissociated tissues were then ground through a 70-µm strainer, and mononuclear cells were obtained by density gradient separation using Ficoll-Hypaque.

### Flow cytometry

PBMC, TIL and NIL were stained with fluorochrome-conjugated mAbs against human CD3, CD4, CD25, CD45RO, CD69, HLA-DR, CCR4, CCR6, CCR7, CD11a, CD62L, CD103, FoxP3, and CTLA-4 (BD PharMingen, San Diego, CA). For intracellular staining, the cells were permeabilized and fixed using Cytofix/Cytoperm (BD PharMingen) according to the manufacturer's instructions. After staining, three- or four-color flow cytometry was performed using LSR II flow cytometer (Becton Dickinson, San Jose, CA), and data were analyzed using Flowjo software (Tree Star, Inc., Ashland, OR).

### Immunohistochemical staining

Paraffin-embedded, formalin-fixed liver tissue was cut into 4-µm sections. Antigen retrieval was preformed via pressure cooking for 10 minutes in citrate buffer (pH 6.0). Antibodies of mouse anti-human FoxP3 (dilution, 1∶200) and rabbit anti-human CCL20 (dilution, 1∶150) (Abcam, Cambridge, UK) were used for the primary antibodies. Diaminobenzidine is used for substrate following counterstaining with hematoxylin for single staining. Double staining was preformed using EnVision™ G|2 Doublestain System (Dako, Glostrup, Denmark) with 2 different Chromogens: DAB^+^ Chromogen (brown color) for FoxP3 and Permanent Red Chromogen (red color) for CCL20. For quantitative analysis of the CCL20 staining, the integrated optical density, which represents the mean optical density multiplied by cell area, was measured by the Image-Pro Plus 6.0 (Media Cybernetics, Baltimore, MD) in 10 high-powered fields as described [36].

### Real-time PCR

RNA was extracted using the RNeasy Mini kit (Qiagen, Hilden, Germany) and synthesized for cDNA using QuantiTech Reverse Transcription kit (Qiagen). Quantitative real-time PCR was conducted in SYBR Green PCR Master Mix (Applied Biosystems, Foster City, CA) using the ABI Prism 7500 Real-time PCR System (Applied Biosystems). The primer sequences listed in Table 4. Samples were run in triplicate, and their relative expression was calculated in the following formula using GAPDH as endogenous controls: 2^−ΔΔCt^.

**Table 4:** Primers for real-time PCR.

Gene	Sense primer (5′-3′)	Antisense primer (5′-3′)
CCL1	TGGATGGGTTCAGAGGCACA	AGGGCAGAAGGAATGGTGTAG
CCL2	CTCATAGCAGCCACCTTCA	GCTTCTTTGGGACACTTGC
CCL3	TCTGGTGACAACCGAGTGGC	CCGATCACAGCCCTGAACAA
CCL4	CCTCGCAACTTTGTGGTAGA	CAGTTCAGTTCCAGGTCATACAC
CCL17	CCAGGGATGCCATCGTTT	GGTGGAGGTCCCAGGTAGTC
CCL19	AAGACTGCTGCCTGTCTGTGA	CTGGATGATGCGTTCTACCC
CCL20	GACATAGCCCAAGAACAGAAA	GACAAGTCCAGTGAGGCACAA
CCL21	TGAAGCCTGAACCCAAGATG	CAGCCATGCAGGGTAGAGC
CCL22	GGAGGCAAAGAGTAGGGTGTAAT	TCAGCCAGAAAGGCATAGATAGA
CXCL9	TTGCTGGTTCTGATTGGAGTG	AAGGTCTTTCAAGGATTGTAGGTG
CXCL12	CCTCTACCTGACACTCCCTT	TGGCACAGACTCAATCCC
GAPDH	CTCTCTGCTCCTCCTGTTCGAC	TGAGCGATGTGGCTCGGCT

### Immunosuppression Assays

CD4^+^CD25^+^ (Treg) and CD4^+^CD25^−^ (Tconv) T cells were separated from PBMC using magnetic beads (purity, >97%; CD4^+^CD25^+^ regulatory T cell isolation kit; Miltenyi Biotec). Purified Tregs (2.5×10^4^/well) were cultured in triplicate with autologous purified Tconv from TIL, NIL, or cPBMC in the presence of 2.5 µg/ml soluble anti-CD3 mAbs (BD PharMingen) plus 5 µg/ml anti-CD28 mAbs (BD PharMingen). The added Treg:Tconv ratio was 0∶1, 0.5∶1, or 1∶1. After 4 days, [^3^H] Thymidine (Amersham, Freiburg, Germany) was added for 16 h (0.5 µCi/well), and then cells were harvested and counted in a scintillation counter.

### Enzyme-linked immunosorbent assay

The concentration of interferon-γ (IFN-γ) in the culture supernatants was determined using enzyme-linked immunosorbent assay kits (eBioscience, San Diego, CA) according to the manufacturer's instructions.

### Chemotaxis assay

CD4^+^CD25^+^ and CD4^+^CD25^−^ T cells were isolated as described above, while chemotaxis assays were performed as previously described [5], [37]. Briefly, 5×10^5^ CD4^+^CD25^+^ or CD4^+^CD25^−^ T cells in a volume of 200 µl were added to the upper wells (insert pore size, 5 µm; Millipore, Billerica, MA). Human chemokines (CCL20, CCL21 and CCL22, 100 ng/ml of each; all from R&D System, Minneapolis, MN), or supernatant of SMMC-7721 cell line (Cell Research Institute of the Chinese Academy of Sciences, Shanghai, China) were added to the lower chamber in a volume of 900 µl. After 4 h at 37°C, cells migrating to the lower chamber were enumerated using a hemocytometer. The SMMC-7721 tumor cell line was cultured in RPMI-1640 medium supplemented with 10% FCS in a humidified 5% CO2 at 37°C. SMMC-7721 supernatant used for chemotaxis assay was collected after 3- to 5-day-old culture. Antibodies to CCL20, CCL21 and CCL22 (R&D System) were added just before the chemotactic experiments at a saturated concentration of 500 ng/ml. Chemotactic index represents the ratio of cells migrated in the presence of CCL20 to the cells migrated spontaneously with medium alone. Assays were performed as triplicates.

### Statistic analysis

All statistical data were analyzed using SPSS, version 16.0. Differences between groups were analyzed using one-way ANOVA, Mann-Whitney U test, or χ2 test where appropriate. Pearson coefficient was computed to assess the association between CCL20 and FoxP3^+^ cells in the tumor environments. Cumulative survival times were calculated by the Kaplan–Meier method and different survival functions between groups were analyzed by the log-rank test. A multivariate Cox proportional hazards regression model was used to identify independent prognostic factors. Data were expressed as means ± S.E.M. Statistical significance was set at *P*<0.05.
